# Yeast cell wall mannan rich fraction modulates bacterial cellular respiration potentiating antibiotic efficacy

**DOI:** 10.1038/s41598-020-78855-5

**Published:** 2020-12-14

**Authors:** Helen Smith, Sharon Grant, Joanne Parker, Richard Murphy

**Affiliations:** Alltech, Summerhill Road, Dunboyne, County Meath Ireland

**Keywords:** Cell biology, Microbiology, Molecular biology

## Abstract

Now more than ever there is a demand to understand the mechanisms surrounding antibiotic resistance and look for alternative ways to impact phenotypic antibiotic outcome. Cellular energetics can be impacted by many bacteriostatic and bactericidal antibiotics, which affect metabolism and energy output, resulting in a reduction of cell growth or induction of cell death respectively. In this study, we provide evidence that a mannan rich fraction (MRF) from the cell wall of *Saccharomyces cerevisiae* modulates growth of antibiotic susceptible and resistant *Escherichia coli* and potentiates bactericidal antibiotic efficiency through modulation of bacterial cellular respiration. The role of MRF in modulating bactericidal impact and cellular metabolic state were assessed in *E. coli* by monitoring microbial growth and by measuring oxygen consumption rate (OCR) and extracellular acidification rate (ECAR) using the Seahorse XFe96 Analyser, respectively. This work further illustrates the link between bacterial susceptibility to antibiotics (phenotypic resistance) and resistance through modulation of bacterial metabolism. This is the first example of yeast MRF enabling collateral sensitivity to antibiotics in vitro and supports the search for alternative strategies to promote animal health without contributing to the growing issue of antimicrobial resistance.

## Introduction

Due to the prevalence of antibiotic resistance and in trepidation of its future global impact, research into ways in which we can support antibiotic restriction is imperative. Central to enhancing our understanding of antibiotic resistance and susceptibility, are investigations into the core mechanisms involved and ways in which we can potentiate beneficial physiological responses without contributing to resistance. Cellular metabolism is a key mitigator in positively or negatively influencing antibiotic activity^1–3^. Antibiotic efficiency has been linked to the bioavailability of molecular oxygen contributing to the electron transport chain^1,4^. Bacteriostatic and bactericidal antibiotics inhibit cell growth or induce cell death, respectively, and are defined phenotypically based on how they target different cellular processes. Specifically, changes to cellular metabolic state have been linked to changes in the phenotypic output of different antibiotic classes^5^. These antibiotics modify cellular metabolic status, in particular via respiration by a number of means, such as formation of ROS and production of harmful hydroxyl radicals^2,6,7^, or by way of $\upbeta$-lactam antibiotics, through increasing metabolic demands through generation of a futile cycle of peptidoglycan synthesis^7,8^. Bacteriostatic antibiotics target translation^8,9^, whereas bactericidal antibiotics are believed to directly influence transcriptional upregulation of metabolic and respiratory genes, and have also been linked to carbon flux through the TCA cycle^2–4,7,8,10^. Both classes of antibiotics are capable of modification of cellular energy dynamics via modulation of these processes^8,11^. With regard to bactericidal antibiotics, previous work has shown the cellular response induces metabolic lethality via ROS formation suggesting that increasing or accelerating respiration may enhance bactericidal activity^8^. Attempts to amplify antibiotic activity through combining antibiotic treatments has shown diverse effects on bacterial survival^7^ and has in some instances had negative implications due to attenuation of bactericidal activity over bacteriostatic lethality^8^. In general, greater efficiency by means of combinatorial antibiotic treatments have displayed additive, synergistic or antagonist effects, in some instances leading to unpredictable permutations. Dietary mannan-oligosaccharides (MOS), as prebiotic zootechnical feed ingredients, are derived from the outer cell wall of yeast and have been extensively studied as a non-pharmaceutical alternative to antibiotic growth promoters^12–14^. MOS, with its ability to bind and limit the colonisation of gut pathogens^15–18^, has proven to be an effective feed supplement supporting immunity and digestion^18,19^. Further refinement of yeast MOS has led to the isolation of a mannose-rich fraction (MRF) with enhanced activities in immune modulation and intestinal health. In the present study we examine how potentiation of cellular respiration enhances antibiotic efficiency.

Previous research determined that bacterial susceptibility to antibiotics is mediated by microbial metabolic regulation^5,15^. By examining the respiration profile of *Escherichia coli,* we gained an insight into how ampicillin, a bactericidal antibiotic, targets growth inhibition and leads to acceleration of cellular respiration. Our data shows that MRF supplemented in the culture media reduced the growth of both sensitive and resistant *E. coli* and positively influenced phenotypic antibiotic outcome when in combination. The data indicates the link between cellular oxygen consumption rate (OCR) and presence of MRF with respect to the bactericidal lethality of ampicillin. Development of antibiotic target-based resistance and the potential unpredictability of antibiotic combinations encourage exploration of non-pharmaceutical alternative sources such as MRF to reduce resistant populations and enhance antibiotic effectiveness. This research is therefore timely with respect to the search for alternate strategies to promote animal health without contributing to antimicrobial resistance.

## Results

### MRF modulates growth of antibiotic susceptible and resistant *E. coli*

**Figure 1:**
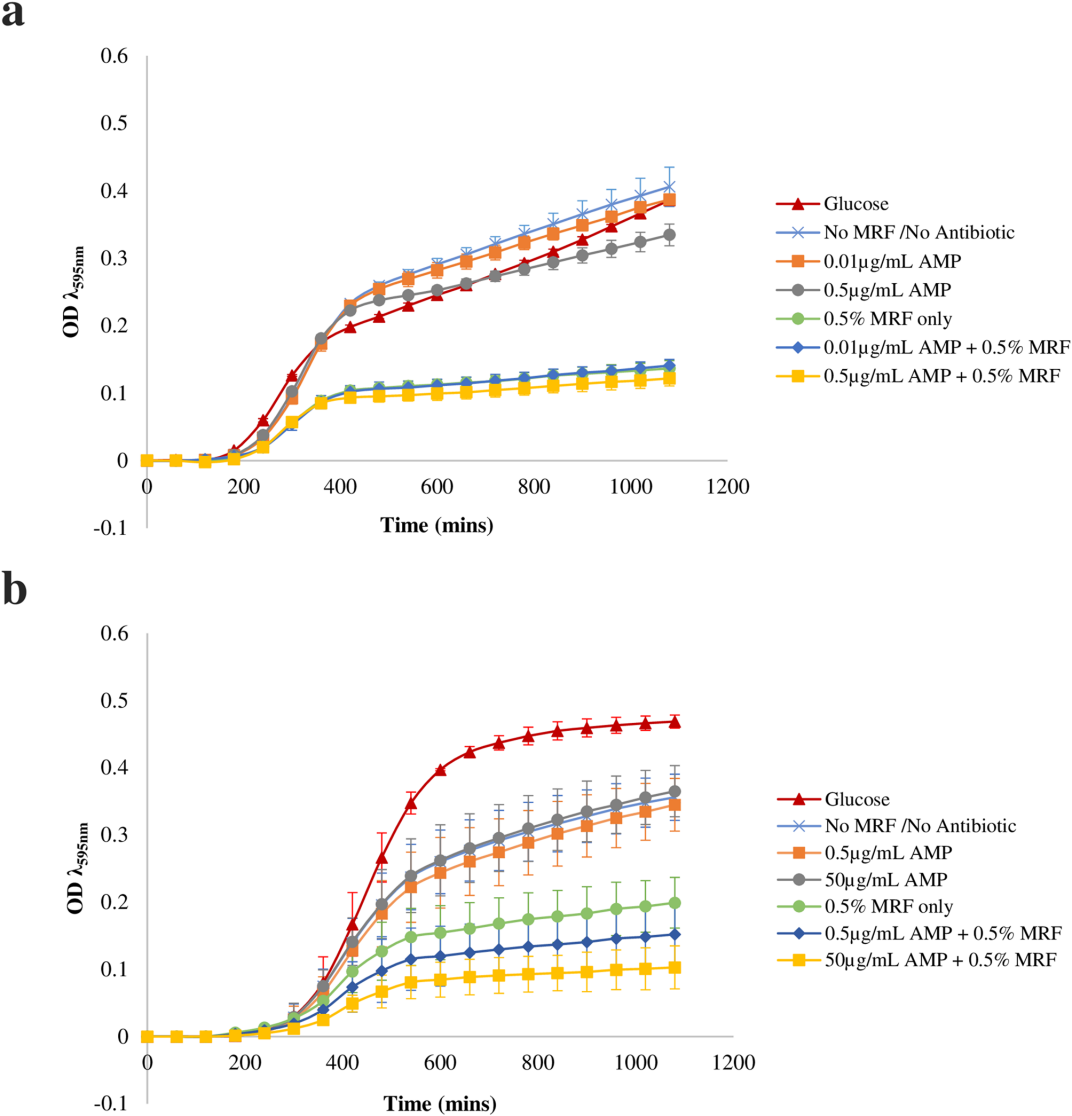
MRF modulates growth of susceptible and resistant *E. coli*. Growth curves of **(a)** antibiotic susceptible *E. coli* and **(b)** antibiotic resistant *E. coli* supplemented and not supplemented with MRF (0.5%), in the presence and absence of ampicillin (AMP). Each value was expressed using mean of triplicate values for each biological replicate (n = 3), standard deviation is represented by error bars.

**Figure 2:**
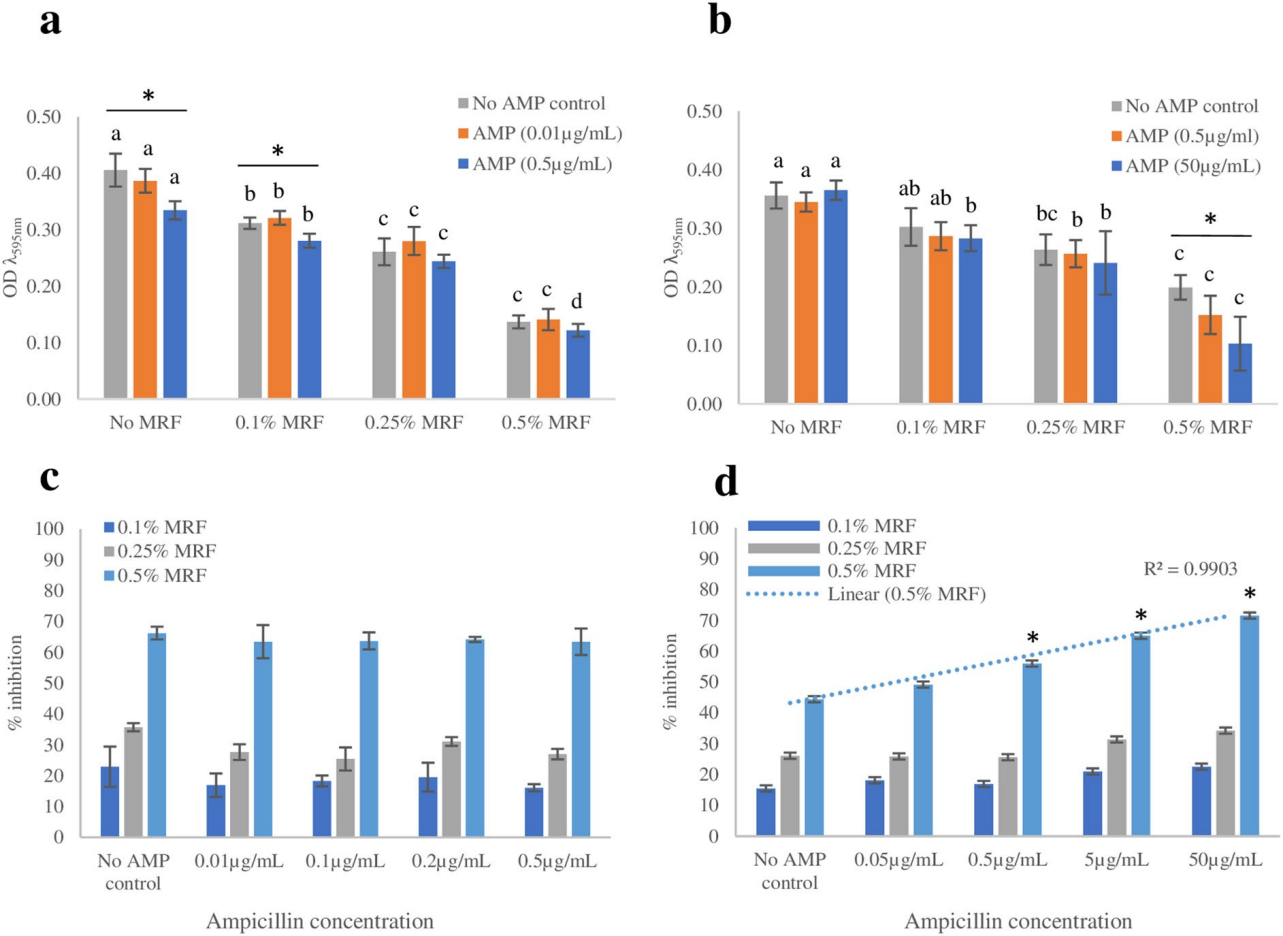
Effect of MRF supplementation on end-point* optical density (OD) and percentage inhibition; **(a)** Final OD of antibiotic susceptible *E. coli*; **(b)** final OD of antibiotic resistant *E. coli*; **(c)** percentage reduction of antibiotic susceptible *E. coli* resulting from MRF supplementation relative to no MRF supplementation; **(d)** percentage reduction of antibiotic resistant *E. coli* resulting from MRF supplementation relative to no MRF supplementation. Each value was expressed using mean of triplicate values for each biological replicate, standard deviation is represented by error bars. Means per AMP concentration that do not share a letter are significantly different to the corresponding ‘No MRF’ relative mean (*p* ≤ 0.05, ANOVA, Fisher-LSD). Values marked with an asterisk [*] are significantly different to the ‘No AMP control’ treatment relative to the corresponding MRF concentration (*p* ≤ 0.05, ANOVA, Fisher-LSD). *End-point = 18 h OD measurement.

In this study, we examine if MRF, a derivative of MOS, potentiates growth of antibiotic susceptible and resistant *E. coli* in conjunction with bactericidal antibiotic treatment. When MRF was supplemented (≥ 0.1% (w/v)) in the media of antibiotic susceptible *E. coli*, with no ampicillin treatment, a significant reduction in final growth measurement (*p* ≤ 0.01) and maximum optical density (OD) (*p* ≤ 0.05) was observed [Figs. 1a and 2a (also Table S1 online)]. The percentage growth inhibition of the susceptible strain was not impacted significantly further due to the combination of MRF and ampicillin treatment (Fig. 2c), nonetheless, increasing MRF supplementation significantly reduced growth (Fig. 2a).

Similarly, when MRF was supplemented (> 0.1% (w/v)) into the growth media of antibiotic resistant *E. coli*, a significant reduction in final growth measurement (*p* ≤ 0.05), maximum OD (*p* ≤ 0.05) and lag time (*p* ≤ 0.05) was observed [Figs. 1b and 2b (also Table S2 online)]. The growth curve, final growth measurement, maximum OD and lag time of the resistant *E. coli* strain was not affected by antibiotic treatment, but instead increasing MRF supplementation, as is illustrated by Figs. 1b and 2b (also Table S2 online). Growth rate is understood to impact the phenotypic outcome of antibiotic susceptibility, a phenomenon first described as early as the discovery of penicillin^5,20^. In this instance, the supplementation of MRF resulted in a significant reduction of cell growth (represented by max OD) (*p* ≤ 0.05) and a greater reduction when supplemented MRF was combined with ampicillin treatment, effectively potentiating the activity of this antibiotic toward the resistant organism (Fig. 1b, also Table S2 online). The presence of MRF (0.1% (w/v)) alone reduced the final growth measurement (OD) of the resistant strain by 15% (Fig. 2d). Growth of resistant cells were reduced by a further 2.5–7.5% when combined with 0.05–50 µg/mL ampicillin treatment, resulting in a maximum total reduction of 22.5% due to the addition of 0.1% (w/v) MRF to the growth media (Fig. 2d). By increasing the presence of MRF to 0.5% (w/v) a 44% reduction in final growth measurement (OD) of the resistant strain was observed (Fig. 2d). Growth of resistant cells were reduced by a further 4–28% when combined with 0.05—50 µg/mL ampicillin treatment, resulting in a maximum total reduction of 72% due to the addition of 0.5% (w/v) MRF to the growth media (Fig. 2d). Thereby, MRF supplementation [0.1–0.5% (w/v)] lead to collateral sensitivity of the resistant organism, demonstrating a linear increase (*r*^2^ = 0.99) in adjunctive sensitivity (*p* ≤ 0.05) when combined with increasing ampicillin treatment (Figs. 1a,b and 2a–d).

It has been shown previously that with an increase of bacterial cell number (and the probability of increased acquired resistance) as well as possible changes to bacterial metabolism leading to phenotypic resistance, the concentration of an antibiotic required to cure experimental infection increases with the duration of the infection ^5,21^. The restriction of bacterial growth observed in this study due to media supplementation with MRF, represents an elegant solution to proliferation of resistant pathogens. Whilst MRF supplementation of the growth media resulted in a considerable growth reduction of antibiotic susceptible and resistant *E. coli*, bacterial susceptibility and resistance to antibiotics depends on more than whether the cells are actively growing or resting, but also relies on their specific metabolic situation^5^. As a critical test of the hypothesis, we examined bacterial redox physiology, via cellular respiration, for any alteration in metabolic respiration or glycolytic flux in response to MRF supplementation in the growth media. This was based on the knowledge that microbial cellular energetics are impacted by bactericidal antibiotics, affecting cellular metabolism and energy output, either via reduction of cell growth or cell death^1,2,4,8^.

### Perturbations of basal respiration rate potentiates bactericidal activity

Previous research determined that oxygen consumption rate (OCR) is dependent on metabolic carbon state^8^. In this study, cellular metabolism was investigated as a marker for physiological bactericidal status. To investigate changes in cellular metabolic respiration and glycolytic flux, OCR (pmol/min) and extracellular acidification rate (ECAR) (mph/min) were measured in real-time using the Seahorse XFe96 Analyser. The assay performed in M9 medium limited *E. coli* growth, resulting in a direct linear measure of respiration in the steady state over time. Unlike other systems which utilise intracellular marker detection or inspect the cell surface, the Seahorse XF analyser measures changes in analytes that are either consumed or excreted by the cell. Antibiotic treatment is injected into the fluid chamber containing a bacterial cell monolayer and M9 media supplemented or not supplemented with MRF; oxygen consumption rate was detected by a steady-state sensor probe.

**Figure 3:**
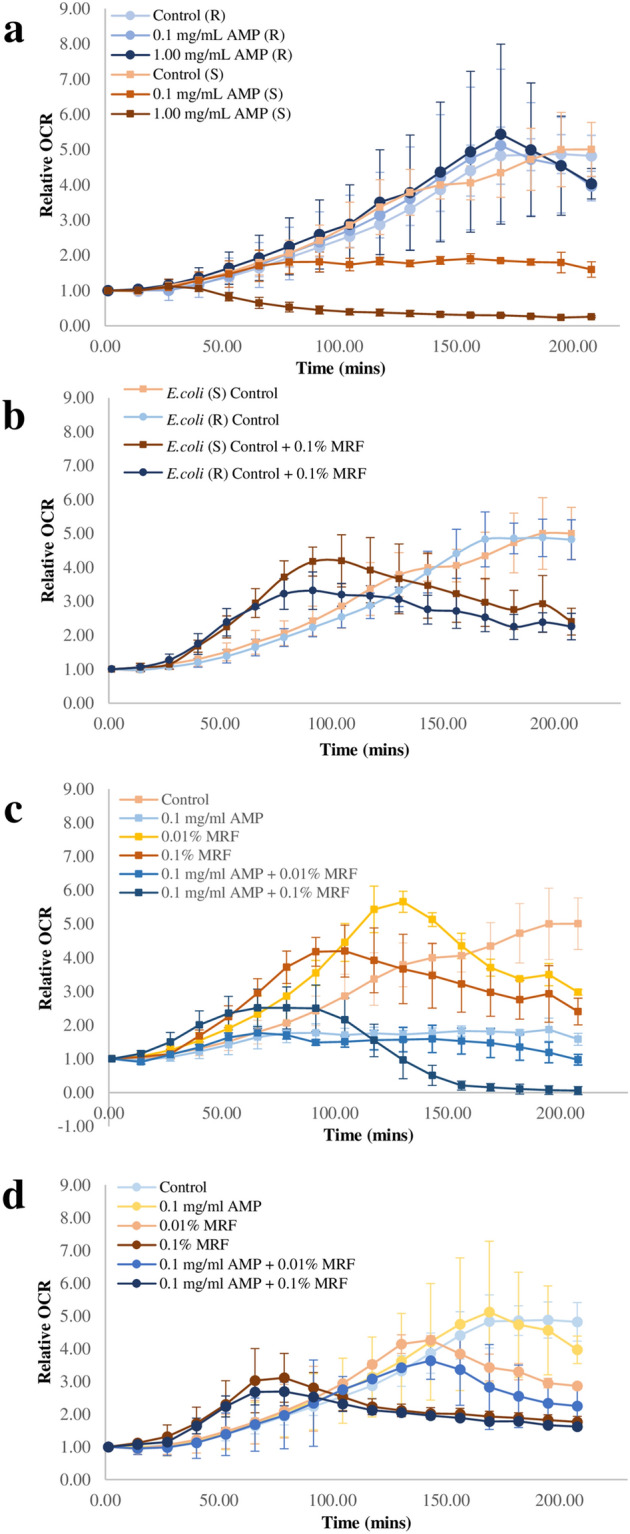
Real time comparison of changes in relative OCR (pmol/min) of antibiotic susceptible and resistant *E. coli*; **(a)** relative OCR (pmol/min) of antibiotic susceptible and resistant *E. coli* in the presence and absence of ampicillin; **(b)** relative OCR (pmol/min) of antibiotic susceptible and resistant *E. coli* with and without MRF supplementation (0.1% (w/v)); **(c)** relative OCR (pmol/min) of antibiotic susceptible *E. coli* with and without MRF supplementation in the presence and absence of ampicillin treatment; **(d)** relative OCR (pmol/min) of antibiotic resistant *E. coli* with and without MRF supplementation in the presence and absence of ampicillin treatment. All data expressed using mean of triplicate values for each biological replicate (n = 3), standard deviation is represented by error bars. (R), *E .coli* resistant to ampicillin; (S), *E. coli* susceptible to ampicillin.

Figure 3a compares real time changes of relative OCR observed with ampicillin treatment of resistant and susceptible *E. coli*. The results show that bacterial susceptibility to ampicillin is impacted by real-time OCR kinetics (Fig. 3a, Fig S1). No change in OCR was observed when the resistant strain was treated with ampicillin, demonstrating a direct link between phenotypic resistance and cellular respiration. Figure 3b shows relative change in OCR over time between antibiotic susceptible and resistant *E. coli* when supplemented and not supplemented with MRF (refer also to Fig S2 online). Supplementation of MRF lead to an acceleration of basal cellular metabolism. When MRF is supplemented into the media, oxygen is consumed at a faster capacity by the cells leading to a subsequent earlier drop in OCR compared to cells not supplemented with MRF. The resistant strain which maintains OCR > 160 min when treated with antibiotics begins to decline ~ 105 min with MRF supplementation, reverting OCR to a similar state to that of the sensitive strain, mimicking OCR of an antibiotic susceptible species (Fig. 3b).

Having established a direct link between antibiotic susceptibility and MRF with respect to cellular metabolism and having shown that MRF supplementation resulted in significant collateral sensitivity of the resistant organism via growth modulation (*p* ≤ 0.05) (Figs. 1b and 2d), we explored the metabolic perturbations of MRF and ampicillin in combination. When susceptible and resistant *E. coli* were treated with ampicillin and MRF in combination, like kinetic growth analysis, an adjunctive response in terms of OCR was observed, revealing a possible link between cellular proliferation and respiration rate. Focusing on MRF supplementation alone, cellular OCR accelerated and peaked sooner than the control culture containing no treatment (Fig. 3b,c). Due to the susceptibility of the non-resistant *E. coli* strain to ampicillin, cellular OCR declines gradually with the addition of this antibiotic, however, an accelerated OCR was also observed with these cells in response to MRF (Fig. 3c). Overall, it is apparent that the metabolic rate of cells increases through exogenous supplementation with MRF.

Increasing MRF (%, w/v) supplementation also altered OCR of antibiotic resistant *E. coli* (Fig. 3d). Ampicillin alone did not impact the OCR of this strain due to its lack of antibiotic susceptibility however, when MRF is added, OCR is accelerated and begins to decline at a faster rate than when no MRF is present in the media. Additionally, an adjunctive change in OCR dynamics relative to the corresponding MRF concentration is observed. When 0.1% MRF is supplemented in the media, combined with and without 0.1 mg/mL ampicillin treatment OCR is accelerated and peaks at a faster capacity. Previous research has shown that elevated basal respiration increases killing by respiration-accelerating bactericidal antibiotics^8^. Here, we demonstrate that this may be the case in the instance of antibiotic resistant organisms also, and MRF is capable of this modulation.

**Figure 4:**
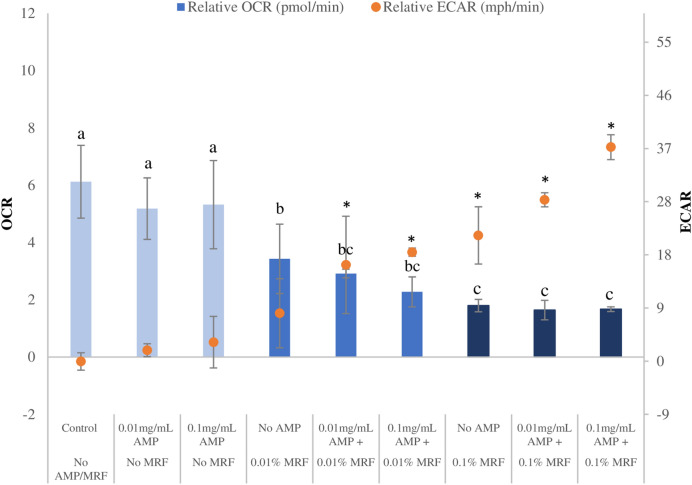
OCR (pmol) and ECAR (mph/min) end-point status following MRF and/or antibiotic treatment. End-point relative OCR (bars) and ECAR (data points) status of antibiotic resistant *E. coli* with and without MRF supplementation combined with and without ampicillin treatment. Each value was expressed using mean of triplicates for each biological replicate (n = 3), standard deviation is represented by error bars. OCR values that do not share a letter are significantly different (*p* ≤ 0.05, ANOVA, Fisher-LSD). ECAR values marked with ‘*’ are significantly different to the control (no AMP/MRF) group (*p* ≤ 0.05, ANOVA, Fisher-LSD).

End-point examination of ECAR status of the resistant strain showed a considerably greater amount of acid secretion when MRF is present in the culture media, greater again when ampicillin was added to MRF supplemented culture media, a demonstration of increased fermentative growth (Fig. 4, also see Fig S3 online)^8^. This indicates that respiratory capacity of *E. coli* cultured in the presence of both MRF and ampicillin is intensified leading to a more stressful/energetic fermentative environment, potentially reducing the growth capacity of the resistant organism more proficiently compared to ampicillin treatment alone.

The data demonstrates the potential of MRF to modulate phenotypic bactericidal activity and resistance, by increasing and elevating cellular respiration and intensifying fermentative growth. Altogether, as well as the ability of MRF to physically adhere to *E. coli* restricting growth, allowing the host to have a competitive advantage for nutrients and partitioning of immunological responses to invasion^13,18^, MRF also demonstrates the potential to modulate antibiotic susceptibility of ampicillin resistant *E. coli*.

### MRF modulates metabolic/bioenergetic phenotypic status

The central parameters to measuring a change in cellular energetics are (1) oxygen, consumption of which is a direct measurement of OCR or aerobic metabolism, and (2) extracellular acidification rate (ECAR), which is a measurement of glycolytic flux. Examining OCR/ECAR ratio, gives an indication of bioenergetic phenotype under certain conditions; different wells/treatments have different basal rates of metabolic activity, representing basal bioenergetic state of a cell. The data presented here demonstrates the shift from basal metabolic rate over time, giving an overview of any change in bioenergetic profile in response to different treatments.

**Figure 5:**
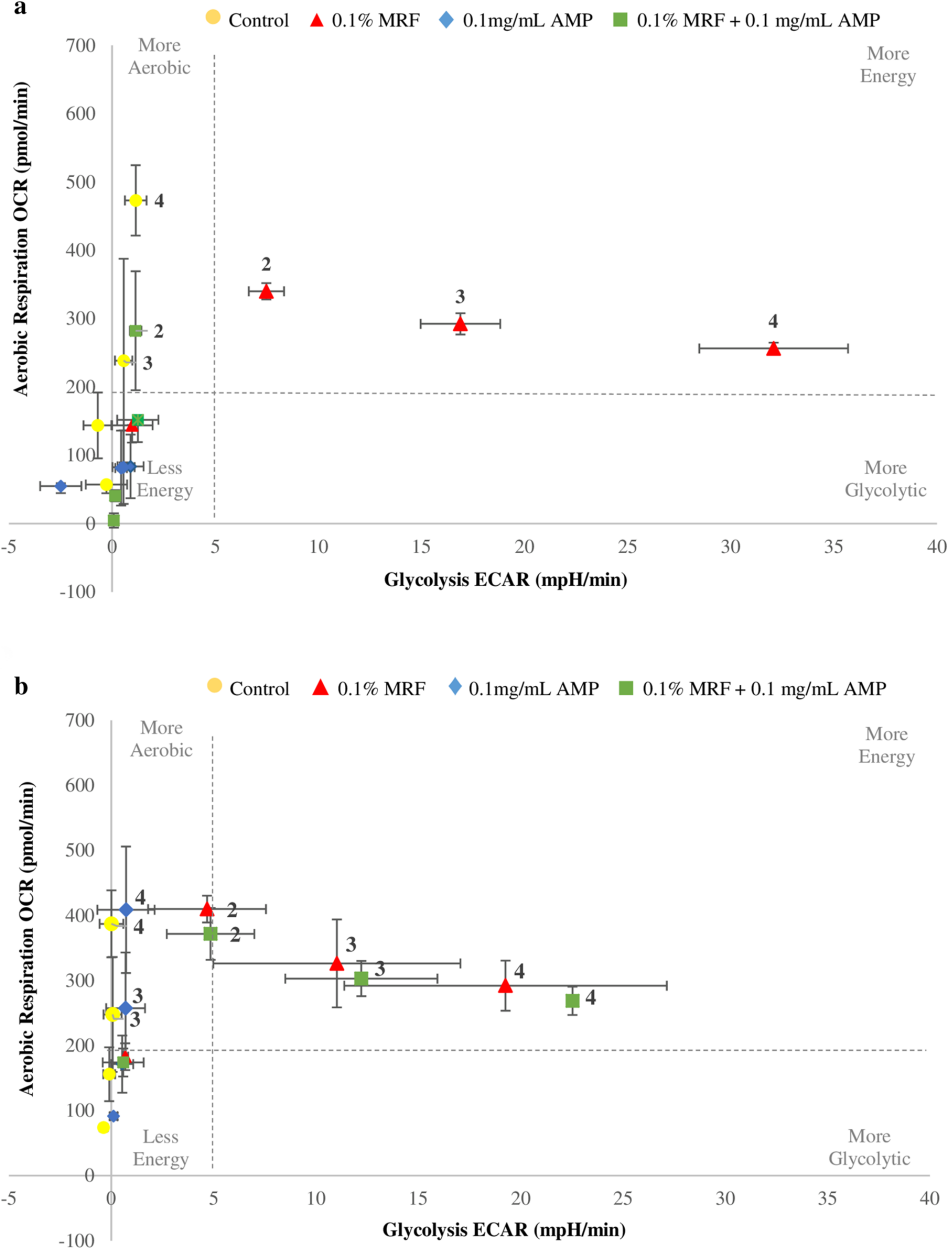
Bioenergetic real time changes in OCR/ECAR ratio of **(a)** antibiotic susceptible *E. coli* and **(b)** antibiotic resistant *E. coli*. Numbered data points represent the following time-points; (1) 15.00 ± 0.80 (basal*), (2) 100.00 ± 4.56, (3) 150.00 ± 0.26, (4) 200.00 ± 1.41 min, respectively. Only data points outside the basal region (bottom left) are labelled with representative time-points to enhance visibility of illustrated data. Each value was expressed using mean of triplicates for each biological replicate (n = 3), standard deviation is represented by error bars. Control refers to no MRF supplementation/AMP treatment; *Basal OCR measured prior to automated antibiotic/M9 injection.

The data showed that untreated susceptible and resistant cells progress to a more aerobic state over time. There was no change in basal cellular energy state when antibiotic susceptible cells were treated with ampicillin, representative of the relationship between antibiotic susceptibility and cellular metabolic state (Fig. 5a). However, resistant cells treated with ampicillin continue to a more aerobic state like the untreated/control cells, demonstrating that antibiotic resistant cellular respiration/bioenergetic profile was not modified by the presence of ampicillin in the media (Fig. 5b). When MRF was supplemented in the media of both susceptible and resistant strains, a dynamic shift in OCR/ECAR ratio is observed (Fig. 5a,b, respectively). MRF resulted in cells being driven to a higher state of metabolic activity. Cells became more aerobic initially before progressively reaching a more glycolytic state over time, simultaneously demonstrating MRF does not negatively impact cellular viability causing cell death, but rather leads to the reduced cellular growth capacity presented previously (Figs. 1 and 2), with modification of cellular respiration capacity a contributing factor. Previous research has shown depletion of metabolic/glycolytic intermediates in *E. coli* result in cellular stress that may appear in the form of growth inhibition^22^. It is hypothesised here that as previous research has shown, glycolytic intermediates, such as glucose-6-phosphate which feeds directly into both glycolysis and the pentose-phosphate pathway, may reroute carbon metabolism to other pathways attempting to restore the balance of central metabolites in stressed cells^22^.

The shift in metabolic activity due to MRF indicates modulation of spare cellular respiratory capacity. These findings underscore that unique metabolic signatures may be critical in strategically targeting resistance to antibiotics and determining whether alternative strategies are viable options to impact phenotypic resistance. Both mammalian cells and bacteriological systems warrant examination of changes to OCR/ECAR ratio as this can suggest modulation of cellular oxidative phosphorylation (OXPHOS) and glycolysis, measurements which may potentially serve as an indicator of a shift in bioenergetic cell metabolic activity. The data demonstrates the significance of cellular energetic state in phenotypic resistance, and how alteration of metabolism may impede phenotypic cell change.

## Discussion

Bacterial metabolism encompasses a vast potential of complex reactions capable of manipulation. In this study MRF, a yeast cell wall fraction modulates basal cellular respiration leading to changes in bacterial metabolism. Perturbation of cellular respiration is a major by-product of antibiotic-target interaction^8^. Previous research has shown exogenous supplementation of specific metabolites can lead to altered metabolism potentiating conventional antibiotics against bacteria in a wide variety of metabolic states^1^. Moreover, emerging evidence suggests that metabolic modulation can overcome antibiotic tolerance/resistance^1^, as was also observed here. Metabolic modulation via exogenous supplementation with MRF can influence antibiotic susceptibility regardless of phenotypic resistance. Combined with its ability to block bacterial lectin, effectively agglutinating to potentially pathogenic/resistant bacteria^23^, the data presented here demonstrates the ability of MRF to modulate growth, cause alteration to cellular respiration and potentiate bioenergetic phenotypic status under specific conditions. MRF supplementation and bactericidal antibiotic treatment was associated with adjunctive accelerated respiratory activity altering basal oxygen consumption. This is indicative of modulation of bacterial redox physiology, influencing the capacity for significant alteration of phenotypic susceptibility or resistance via cellular metabolomics^1,4,8^.

Traditional overuse or misuse of antibiotics has led to the evolution of widespread antibiotic resistance. Consequently, alternative strategies are required to reduce antibiotic load on-farm while supporting restriction of antibiotics in animals to therapeutic use. Modulation of cellular proliferation and metabolism of the gut microflora by various antibiotics is non-specific. By fully understanding the molecular epidemiology associated with antibiotic resistant pathogens it is possible to modulate metabolic mechanisms to enhance susceptibility of resistant pathogens towards antimicrobial agents currently in use without impacting emergence or evolution of resistance. By increasing our knowledge of the interaction between MRF and antibiotics it may be possible to strategically reduce antibiotic use naturally. Further investigation is required to examine feed additives and specific cellular metabolic determinants and their link with metabolic networks to improve antibiotic therapeutic strategies. It is hoped that the current study complements and warrants further exploration toward development of strategies to combat antimicrobial resistance.

## Methods

### Bacteria and yeast cell wall preparations

One Shot TOP10 chemically competent *E. coli* (Invitrogen) was used as the host strain for transformation. Recombinant *E. coli* transformed using pBR322 (Invitrogen) was cultured in LB medium at 37 °C with addition of 100 µg mL^−1^ of ampicillin (AMP) and 30 µg mL^−1^ tetracycline (TET). The recombinant strain was used as the resistant test organism throughout. Yeast mannan-rich fraction (MRF) from the cell wall of *S. cerevisiae* was provided by Alltech Biotechnology (Alltech Biotechnology, Nicholasville, KY). For determination of bacterial kinetic growth and respiratory analysis, the yeast fraction (0.1–0.5%) was ground and added to either fresh MHB or M9 minimal media (supplemented with 0.2% casamino acids and 10 mM glucose^8^), respectively. To ensure homogenous mixing, this suspension was then sonicated using an HTU Soni 130 ultrasonic processor at a power setting of 130 Watts for 3 min on ice. Aliquots were stored at 4 °C prior to use or at − 70 °C for long term storage. All antibiotics and purified glucose [0.1% (w/v)] were obtained from Sigma.

### Inoculum preparation and storage

Frozen stocks of bacteria [5% (v/v)] in 70% glycerol were stored at − 70 °C in LB medium. Working plates were prepared by transferring a single colony using spread plate technique to fresh agar and incubating overnight. Streaked plates were stored at 4 °C for up to six months. Fresh inoculum was prepared by isolating a single colony from working plates into approximately 25 mL of LB broth and grown overnight at 37 °C. Optical density (OD) at λ_595nm_ was adjusted accordingly by spectrophotometer (Shimadzu UV-1601PC).

### Growth and maintenance of recombinant *E. coli*

LB medium (8 g L^−1^) was sterilised at 105 °C for 30 min. If agar was required, 15 g L^−1^ was added before autoclaving. To facilitate the growth of *E. coli,* medium was cooled to approximately 55 °C, after which AMP and TET were added to a final concentration of 100 µg mL^−1^ and 30 µg mL^−1^, respectively. Agar plates were stored in the dark at 4 °C and were warmed to 37 °C prior to use.

### Kinetic microplate growth analysis

MRF [0–0.5% (w/v)] was prepared in MHB broth. Aliquots of 200 μL per well and analysed in triplicate at OD λ_595nm_ in a sterile 96 well flat-bottom microtitre plate. A serial dilution of the appropriate antibiotic was prepared separately and added per well (10 μL). To this, 20 μL aliquots of the test organism (adjusted to 0.01 OD at λ_595nm_ (approx. 1 × 10^–8^ cfu mL^−1^)) was added to each well. A reference control (RC) with no antibiotic was included per MRF concentration. The plate was then incubated under appropriate growth conditions; in this case 37 °C. The OD was read every hour following a 5 s medium shake for a total of 18 h. Optical density measurements following 18 h incubation were taken and compared to the reference control. Glucose (0.1% (w/v)) was used as a positive control. Replicate analysis was conducted using a Tecan Infinite M200 Pro microplate reader. The growth rates in each well were estimated using the same strain in identical media and the experiment was repeated three times independently, with three replicates per sample.

### Bacterial respiration

Bacterial respiration expressed as OCR was quantified using an XFe96 Extracellular Flux Analyzer (Seahorse Bioscience). Overnight cultures of susceptible and resistant *E. coli* cells were diluted into fresh LB media and grown to an OD λ_595nm_ of ∼ 0.3 at 37 °C. Cells were centrifuged at 4000 rpm for 5 min then washed with fresh M9 media. This process was repeated twice. Cells were diluted to 0.015 with fresh M9 minimal medium or M9 supplemented with MRF (0.01 or 0.1% (w/v), respectively). For OCR measurements, 90 μL of diluted cells were seeded onto XF cell culture microplates, precoated with 15 µL poly-d-lysine (PDL) (Sigma) and centrifuged for 10 min at 1400 rpm to enable attachment. Cellular respiration was quantified following addition of 90 μL of fresh M9 media to each well. To assure uniform cellular seeding, basal OCR was measured for two cycles before automated antibiotic injection (20 µL) and then quantified every 6 min for the duration of the experiment post-treatment. To examine cellular energetic state, OCR and ECAR measurements were used to form energy map diagrams.

### Statistical analysis

Statistical evaluation of growth curve data was assessed using GrowthRate (GR) software, downloadable from https://sourceforge.net/projects/growthrates/^24^. GrowthRate reports correlation coefficient, R, of the best fit line to the natural log of the optical density (ln(OD)) over time for each culture. For each set of replicate cultures, GR reports the mean growth rate, the mean R, max OD and lag time (Tables S1 and S2).

Statistical analyses of results were performed using Minitab statistical software package version 16 (Coventry U.K.). One-way analysis of variance (ANOVA) and Fishers multiple comparisons were carried out to test any significant differences among means, where the confidence level was set at 95%. One-way analysis of variance (ANOVA) and Dunnett’s comparisons were carried out to test any significant differences between each test mean and a control mean, where the confidence level was set at 95%. Significant levels were defined using *p* ≤ 0.05.

## Supplementary Information

Supplementary Information.
