# Supplementary material for: Yeast cell wall mannan rich fraction modulates bacterial cellular respiration potentiating antibiotic efficacy
Source: Sci Rep. 2020 Dec 14;10:21880. doi: 10.1038/s41598-020-78855-5 (PMC7736855; doi:10.1038/s41598-020-78855-5)
Supplement: Supplementary file 1 — Supplementary Information. [file 41598_2020_78855_MOESM1_ESM.pdf]

Supplementary Information (SI)

This file contains supplementary information for manuscript entitled “Yeast cell wall mannan rich fraction modulates bacterial cellular respiration potentiating antibiotic efficacy”.

Smith H.*, Grant S., Parker J., Murphy, R.

Helen Smith*
Email: hsmith@alltech.com

This PDF file includes:

Figures S1 to S3
Tables S1 to S2
SI References

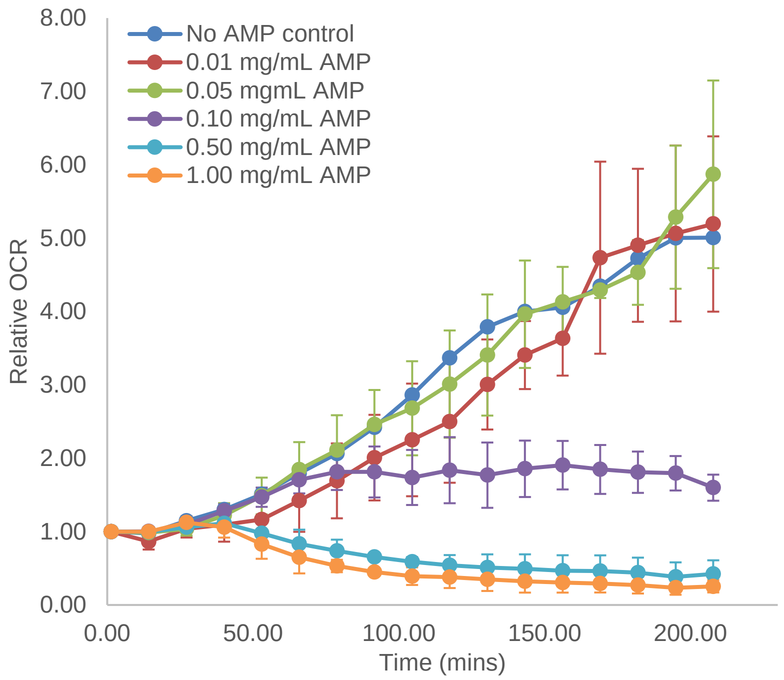

Fig. S1a

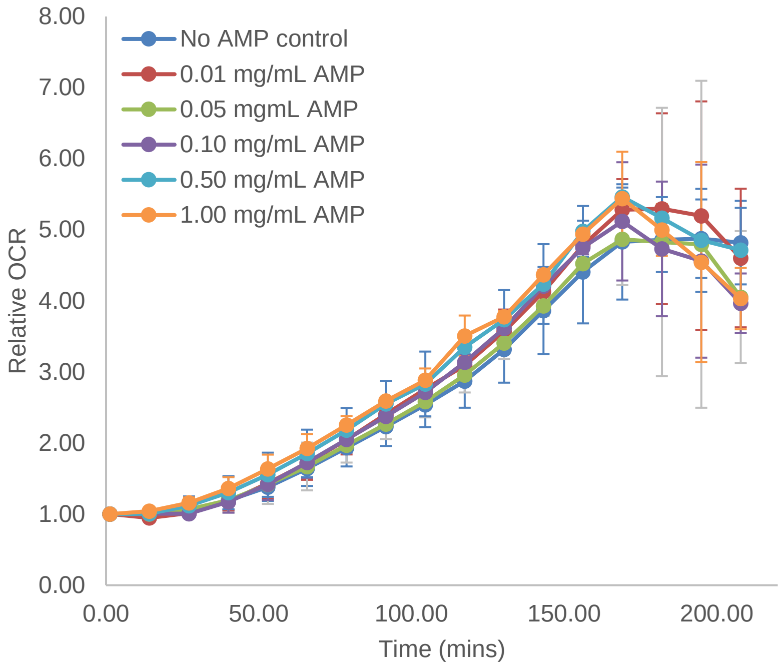

Fig. S1b

Fig. S1. Effect of ampicillin on relative oxygen consumption rate (OCR) (pmol/min) of antibiotic susceptible and *resistant E. coli*. Real-time changes in OCR of **[a]** antibiotic susceptible and **[b]** antibiotic resistant *E. coli* when treated with increasing concentrations of ampicillin (0.01 – 1.0 mg/mL). Each value was expressed using mean of triplicates for each biological replicate (n = 3), standard deviation is represented by error bars.

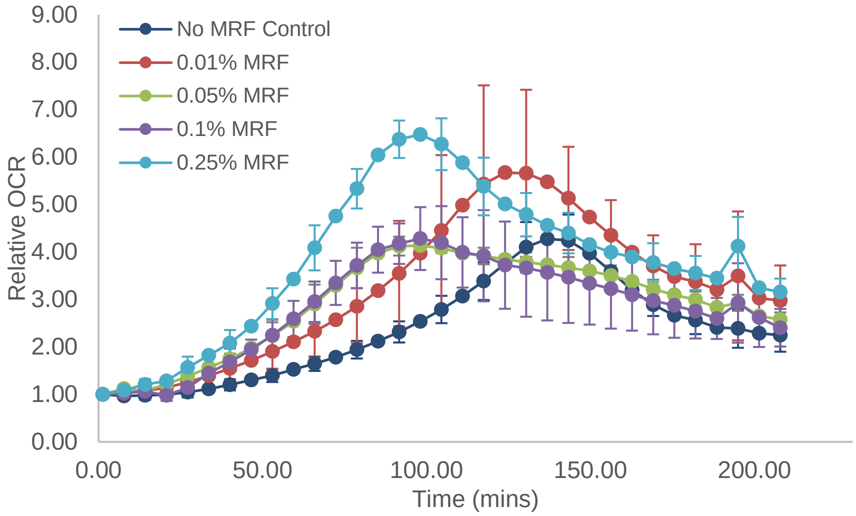

Fig. S2a

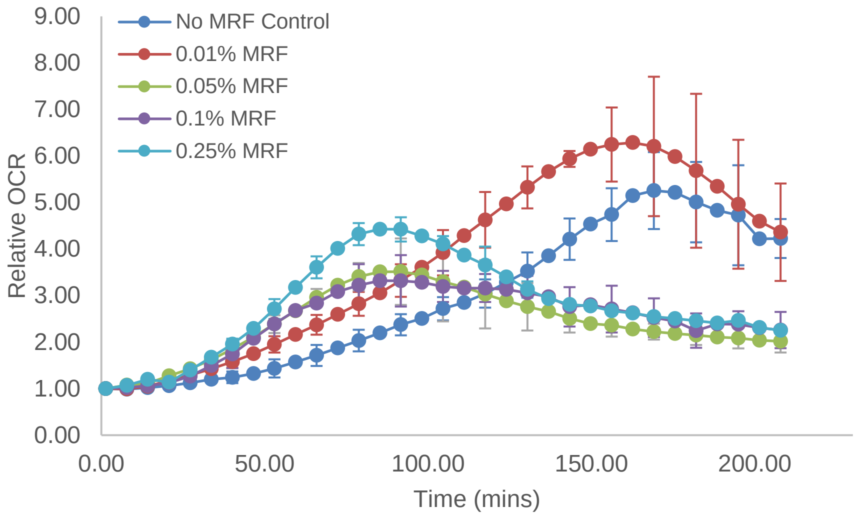

Fig. S2b

Fig. S2. MRF supplementation (0.01 – 0.25%, w/v) potentiates bacterial cellular respiration. Real-time changes in oxygen consumption rate (OCR) of **[a]** antibiotic susceptible and **[b]** antibiotic resistant *E. coli* when treated with increasing concentration of MRF (0.01 – 0.25 %, w/v). Each value was expressed using mean of triplicate values for each biological replicate (n = 3), standard deviation is represented by error bars.

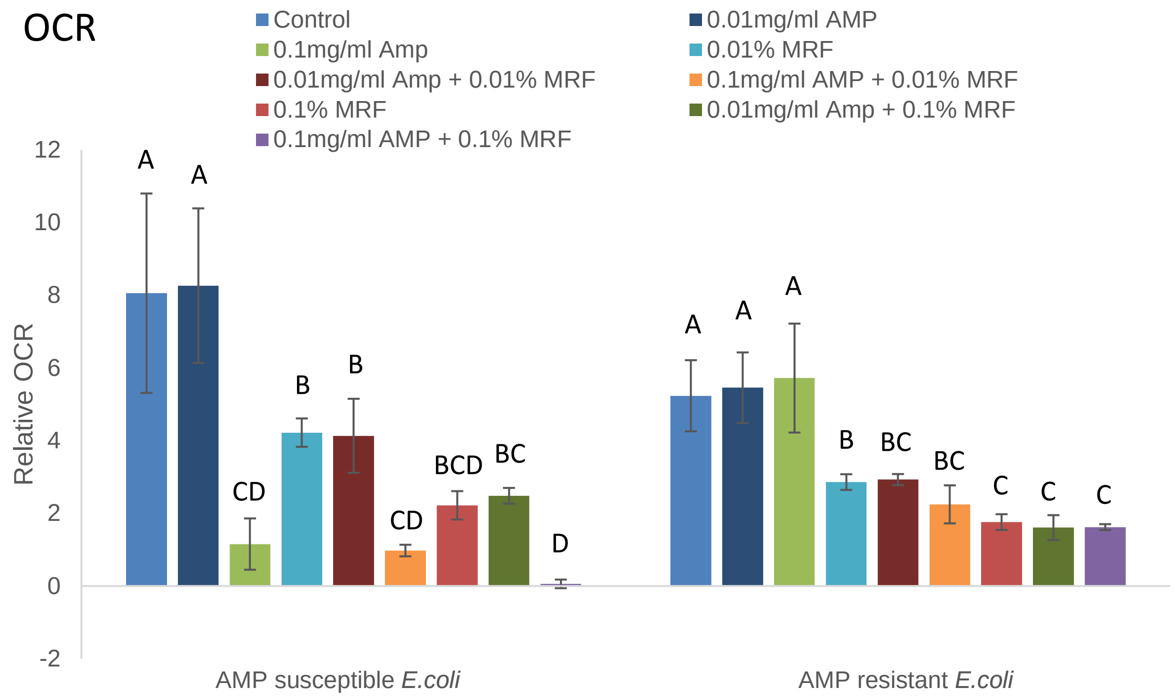

Fig. S3a

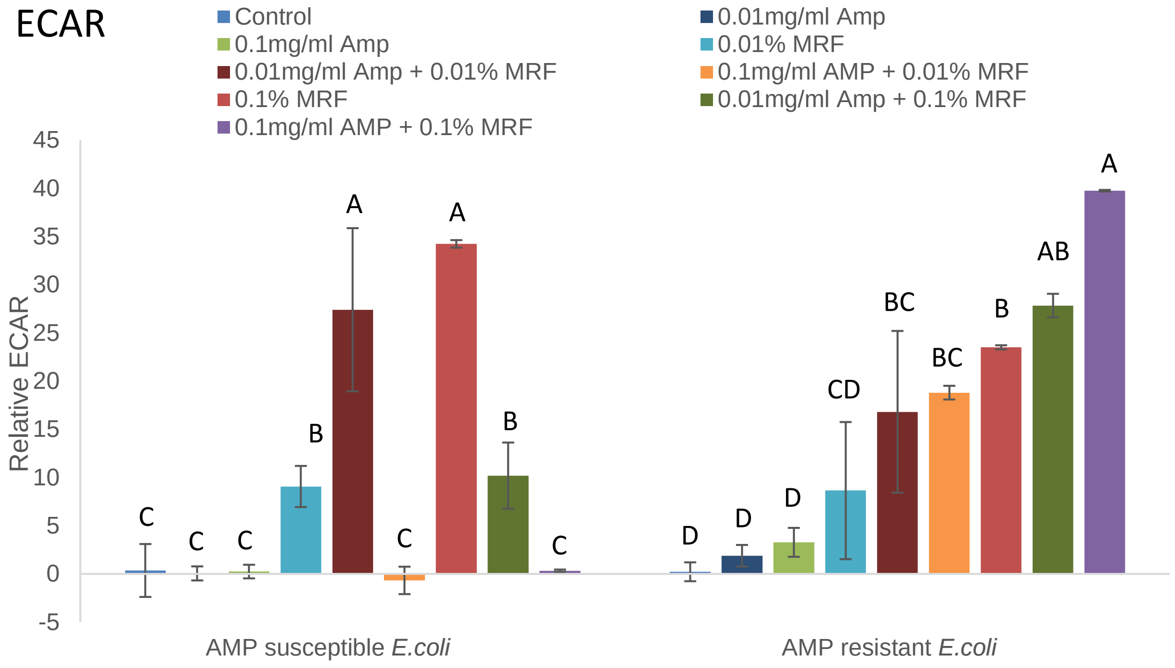

Fig.S3b

Fig. S3. Relative oxygen consumption rate (OCR) (pmol/min) and extracellular acidification rate (ECAR) (mph/min) end-point status. Comparison of **[a]** relative OCR (pmol/min) and **[b]** ECAR (mph/min) end-point measurements between antibiotic susceptible and resistant *E. coli* supplemented and not supplemented with MRF, treated and not treated with ampicillin. Each value was expressed using mean of triplicate values for each biological replicate (n = 3), standard deviation is represented by error bars. Means that do not share a letter are significantly different ($p \leq 0.01$, ANOVA, Fisher-LSD).

Table S1. Growth curve data of antibiotic susceptible *E.coli* generated using GrowthRate software [1]**.**

		No AMP	0.01µgmL^{-1}	0.1 µgmL^{-1}	0.2 µgmL^{-1}	0.5 µgmL^{-1}
Rate	No MRF	0.044 ± 0.000 [a]	0.044 ± 0.000 [a]	0.044 ± 0.000 [a]	0.042 ± 0.003 [a]	0.044 ± 0.000 [a]
	0.1% MRF	0.038 ± 0.001 [b]	0.039 ± 0.000 [b]	0.038 ± 0.001 [b]	0.038 ± 0.000 [b]	0.039 ± 0.000 [c]
	0.25% MRF	0.039 ± 0.000 [b]	0.039 ± 0.000 [b]	0.039 ± 0.001 [ab]	0.039 ± 0.001 [ab]	0.040 ± 0.000 [bc*]
	0.5% MRF	0.038 ± 0.003 [b]	0.036 ± 0.001 [c]	0.038 ± 0.005 [b]	0.041 ± 0.000 [ab]	0.042 ± 0.002 [b]
R	No MRF	0.95 ± 0.005	0.94 ± 0.000	0.94 ± 0.005	0.95 ± 0.012	0.94 ± 0.006
	0.1% MRF	0.94 ± 0.014	0.94 ± 0.014	0.94 ± 0.013	0.95 ± 0.016	0.95 ± 0.001
	0.25% MRF	0.92 ± 0.000	0.92 ± 0.004	0.93 ± 0.007	0.93 ± 0.006	0.94 ± 0.005
	0.5% MRF	0.96 ± 0.003	0.96 ± 0.001	0.95 ± 0.024	0.95 ± 0.008	0.94 ± 0.021
Lag Time	No MRF	133.63 ± 1.15 [a]	132.30 ± 0.00 [a]	132.63 ± 0.98 [a]	114.50 ± 31.35 [a]	132.77 ± 1.27 [a]
	0.1% MRF	73.10 ± 2.94 [b]	73.03 ± 2.89 [c]	73.07 ± 2.66 [ab]	75.87 ± 4.04 [b]	74.83 ± 0.23 [b]
	0.25% MRF	68.83 ± 0.06 [b]	68.47 ± 0.75 [d]	69.40 ± 1.39 [b]	70.77 ± 1.15 [b*]	70.90 ± 1.04 [b*]
	0.5% MRF	97.60 ± 36.20 [b]	76.23 ± 0.29 [b]	113.90 ± 64.26 [ab]	133.73 ± 1.96 [a]	151.50 ± 30.6 6 [a*]
Max OD	No MRF	0.404 ± 0.029[a]	0.391 ± 0.014 [a]	0.383 ± 0.001 [a]	0.391 ± 0.013 [a]	0.341 ± 0.006 [a*]
	0.1% MRF	0.317 ± 0.009 [b]	0.328 ± 0.005 [b]	0.317 ± 0.001 [b]	0.314 ± 0.008 [b*]	0.284 ± 0.008 [b*]
	0.25% MRF	0.261 ± 0.023 [c]	0.283 ± 0.021 [c]	0.294 ± 0.003 [c*]	0.271 ± 0.015 [c]	0.245 ± 0.010 [c*]
	0.5% MRF	0.141 ± 0.004 [d]	0.151 ± 0.018 [d]	0.136 ± 0.008 [d]	0.138 ± 0.001 [d]	0.114 ± 0.010 [d*]

GrowthRate software output (n = 3) for antibiotic susceptible *E. coli* treated with ampicillin, supplemented with and without MRF. Means per AMP concentration that do not share a letter are significantly different. Values marked with an asterisk [*] are significantly different to the control 'No AMP' treatment group for the corresponding MRF concentration ($p \leq 0.05$, ANOVA, Fisher-LSD).

Table S2. Growth curve data of antibiotic resistant *E.coli* generated using GrowthRate software [1]**.**

		No AMP	0.05μgmL^{-1}	0.5 μgmL^{-1}	5 μgmL^{-1}	50 μgmL^{-1}
Rate	No MRF	0.042 ± 0.001 ^{a}	0.040 ± 0.003 ^{a}	0.042 ± 0.001 ^{a}	0.040 ± 0.003 ^{a}	0.040 ± 0.003 ^{a}
	0.1% MRF	0.034 ± 0.001 ^{b}	0.034 ± 0.002 ^{b}	0.033 ± 0.004 ^{b}	0.035 ± 0.002 ^{ab}	0.033 ± 0.004 ^{b}
	0.25% MRF	0.033 ± 0.004 ^{b}	0.034 ± 0.002 ^{b}	0.034 ± 0.002 ^{b}	0.034 ± 0.004 ^{b}	0.035 ± 0.003 ^{ab}
	0.5% MRF	0.036 ± 0.002 ^{b}	0.035 ± 0.001 ^{b}	0.034 ± 0.001 ^{b}	0.033 ± 0.002 ^{b}	0.036 ± 0.003 ^{ab}
R	No MRF	0.95 ± 0.009	0.95 ± 0.006	0.95 ± 0.010	0.95 ± 0.008	0.95 ± 0.008
	0.1% MRF	0.93 ± 0.008	0.95 ± 0.007	0.93 ± 0.024	0.93 ± 0.016	0.93 ± 0.015
	0.25% MRF	0.92 ± 0.017	0.91 ± 0.022	0.92 ± 0.014	0.92 ± 0.005	0.90 ± 0.034
	0.5% MRF	0.90 ± 0.008	0.92 ± 0.006	0.92 ± 0.026	0.91 ± 0.047	0.93 ± 0.015
Lag Time	No MRF	153.80 ± 34.10 ^{a}	135.03 ± 58.51 ^{a}	153.23 ± 34.52 ^{a}	134.80 ± 57.95 ^{a}	135.57 ± 57.85 ^{a}
	0.1% MRF	70.93 ± 1.62 ^{b}	73.73 ± 1.55 ^{b}	68.97 ± 6.69 ^{b}	69.63 ± 3.11 ^{a}	67.67 ± 4.92 ^{a}
	0.25% MRF	66.67 ± 5.60 ^{b}	66.60 ± 2.91 ^{b}	68.93 ± 2.27 ^{b}	66.30 ± 3.27 ^{a}	103.00 ± 58.90 ^{a}
	0.5% MRF	65.73 ± 0.75 ^{b}	68.83 ± 1.12 ^{b}	69.23 ± 4.83 ^{b}	103.33 ± 56.30 ^{a}	130.90 ± 62.00 ^{a}
Max OD	No MRF	0.361 ± 0.034 ^{a}	0.360 ± 0.039 ^{a}	0.350 ± 0.055 ^{a}	0.368 ± 0.045 ^{a}	0.370 ± 0.038 ^{a}
	0.1% MRF	0.307 ± 0.069 ^{ab}	0.296 ± 0.058 ^{ab}	0.291 ± 0.052 ^{ab}	0.292 ± 0.051 ^{ab}	0.287 ± 0.047 ^{b}
	0.25% MRF	0.267 ± 0.045 ^{bc}	0.268 ± 0.047 ^{bc}	0.260 ± 0.044 ^{b}	0.253 ± 0.044^{ab}	0.244 ± 0.046 ^{b}
	0.5% MRF	0.201 ± 0.038 ^{c}	0.184 ± 0.042 ^{c}	0.153 ± 0.028 ^{c}	0.128 ± 0.019 ^{c*}	0.104 ± 0.032 ^{c*}

GrowthRate software output (n = 3) for antibiotic resistant *E. coli* treated with ampicillin, supplemented with and without MRF. Means per AMP concentration that do not share a letter are significantly different. Values marked with an asterisk [*] are significantly different to the control 'No AMP' treatment group for the corresponding MRF concentration ($p \leq 0.05$, ANOVA, Fisher-LSD).

SI References

1 Hall, B.G., *et al.* Growth rates made easy. *Mol Biol Evol.* **31,** 232-238 (2014).
